# SARS-CoV-2 viral proteins NSP1 and NSP13 inhibit interferon activation through distinct mechanisms

**DOI:** 10.1371/journal.pone.0253089

**Published:** 2021-06-24

**Authors:** Christine Vazquez, Sydnie E. Swanson, Seble G. Negatu, Mark Dittmar, Jesse Miller, Holly R. Ramage, Sara Cherry, Kellie A. Jurado

**Affiliations:** 1 Department of Microbiology, University of Pennsylvania Perelman School of Medicine, Philadelphia, Pennsylvania, United States of America; 2 Department Pathology and Laboratory Medicine, University of Pennsylvania Perelman School of Medicine, Philadelphia, Pennsylvania, United States of America; 3 Department of Microbiology and Immunology, Thomas Jefferson University Sidney Kimmel Medical College, Philadelphia, Pennsylvania, United States of America; University of Hong Kong, HONG KONG

## Abstract

Severe acute respiratory syndrome coronavirus 2 (SARS-CoV-2) has caused a devastating global pandemic, infecting over 43 million people and claiming over 1 million lives, with these numbers increasing daily. Therefore, there is urgent need to understand the molecular mechanisms governing SARS-CoV-2 pathogenesis, immune evasion, and disease progression. Here, we show that SARS-CoV-2 can block IRF3 and NF-κB activation early during virus infection. We also identify that the SARS-CoV-2 viral proteins NSP1 and NSP13 can block interferon activation via distinct mechanisms. NSP1 antagonizes interferon signaling by suppressing host mRNA translation, while NSP13 downregulates interferon and NF-κB promoter signaling by limiting TBK1 and IRF3 activation, as phospho-TBK1 and phospho-IRF3 protein levels are reduced with increasing levels of NSP13 protein expression. NSP13 can also reduce NF-κB activation by both limiting NF-κB phosphorylation and nuclear translocation. Last, we also show that NSP13 binds to TBK1 and downregulates IFIT1 protein expression. Collectively, these data illustrate that SARS-CoV-2 bypasses multiple innate immune activation pathways through distinct mechanisms.

## Introduction

Severe acute respiratory syndrome coronavirus 2 (SARS-CoV-2) is a novel human coronavirus that emerged in Wuhan, China in late 2019 [1]. It is a single-stranded, positive-sense RNA virus that belongs to the *Coronaviridae* family of viruses. SARS-CoV-2 is the third *Coronaviridae* virus to emerge into humans and become a public health concern in just the last two decades; following SARS-CoV in 2003 and Middle East respiratory syndrome coronavirus (MERS-CoV) in 2012 [2, 3]. Infection with SARS-CoV-2 causes Coronavirus Disease-2019 (COVID-19) [4]. Despite drastic efforts to mitigate transmission, SARS-CoV-2 has spread globally and caused COVID-19-related deaths worldwide. There is an urgent need for mechanistic insight into the initial molecular events and host-SARS-CoV-2 interactions that progress toward the inflammatory disease state of COVID-19.

The innate immune antiviral program provides the first barrier of protection against viral pathogens. Upon RNA virus infection, viral pathogen associated molecular patterns (PAMPs), such as viral RNA, are sensed by cytosolic pattern recognition receptors, including RIG-I, MDA5, and TLR family members [5]. RIG-I-viral RNA binding initiates a downstream signaling cascade through a central innate immune adaptor protein, MAVS. Once MAVS is activated, the kinases TBK1 and IKKε phosphorylate IRF3, leading to its nuclear translocation and transcriptional induction of interferon-beta (IFN-β) [6, 7]. Secreted IFN-β binds to the IFN-ɑ/β receptors (IFNAR) on neighboring cells, initiating the interferon response and signaling through the JAK/STAT receptor kinases to produce interferon-stimulated genes (ISGs) [8]. In addition to its role in IFN induction in the RIG-I signaling pathway, TBK1 functions in several other pathways, including inflammation, autophagy, and NF-κB signaling [9]. TBK1 helps facilitate NF-κB signaling by forming a complex with the signaling molecule NEMO and the kinases IKKɑ and IKKβ. This complex of molecules initiates downstream phosphorylation and ubiquitination events that results in the degradation of the inhibitor of κB (IκB) and subsequent dimerization and translocation of NF-κB [10]. The extensive crosstalk between signaling events elegantly functions to produce ISGs that act to limit viral replication and spread.

RNA viruses, including coronaviruses, have evolved numerous strategies to bypass antiviral signaling programs, including inactivation of viral RNA sensors, transcription factor induction, or through prevention of innate immune protein-protein interactions. For example, MERS-CoV can inhibit PKR, RIG-I, or MDA5 activation through the actions of its accessory protein, protein 4a [11, 12]. SARS-CoV is also able to block IFN signaling through preventing IRF3 dimerization and phosphorylation [13]. TBK1 signaling is a common target for viruses to evade host immune responses. For example, the MERS-CoV protein ORF4b prevents TBK1 interactions with downstream effectors by binding to TBK1 [14, 15]. Additionally, the SARS-CoV M protein can block IRF3 activation through preventing interaction with TBK1 [16]. Coronaviruses have also been found to impede host mRNA translation to reduce IFN signaling. Previous studies have identified a role of the MERS-CoV and SARS-CoV nonstructural protein NSP1 in blocking interferon signaling through inhibiting host protein translation [17–21]. Here, we identify that SARS-CoV-2 blocks immune activation during infection and demonstrate a role for the SARS-CoV-2 proteins NSP1 and NSP13 in blocking IFN and NF-κB activation. Our study corroborates and is wholly consistent with the recently published studies that have identified immune regulatory roles of the SARS-CoV-2 proteins NSP1 and NSP13 [21–26].

## Materials and methods

### Cell culture

HeLa cells [27] were grown at 37°C with 5% CO_2_ in Dulbecco’s modification of eagle’s medium (DMEM; Gibco) supplemented with 10% fetal bovine serum (FBS; HyClone), 1% penicillin-streptomycin (Gibco), and 1% Gluta-Max (Gibco). Calu-3 cells (ATCC; HTB-55) were grown at 37°C with 5% CO_2_ in minimum essential medium (MEM; Gibco) supplemented with 10% FBS (Hyclone), 1% penicillin-streptomycin (Gibco), 1% Gluta-Max (Gibco). 293T cells (ATCC; CRL-3216) were grown in DMEM supplemented with 10% FBS (Hyclone) and 1% penicillin-streptomycin (Gibco). All cells were tested and found to be *Mycoplasma*-free at the Cell Center Services Facility (University of Pennsylvania).

### Virus infections

SARS-Related Coronavirus 2, isolate USA-WA1/2020, NR-52281 was deposited by the Centers for Disease Control and Prevention and obtained through BEI Resources, NIAID, NIH. SARS CoV-2 virus stocks were propagated in Vero cells. All work with SARS CoV-2 infectious virus (MOI 0.5) was performed in Biosafety Level 3 laboratory and approved by the Penn Institutional Biosafety Committee and Environmental Health and Safety. SeV (Cantell strain; Charles River Laboratories) infections were undertaken in complete media at indicated time points.

### Plasmids and transfections

The following pLVX- SARS-CoV-2-Strep plasmids obtained from Addgene were used in this study: NSP1 (No 141367), NSP2 (No 141368), NSP4 (No 141369), NSP5 (No 141371), NSP7 (No 141373), NSP8 (No 141374), NSP9 (No 141375), NSP10 (No 141376), NSP11 (No 141377), NSP12 (No 141378), NSP13 (No 141379), NSP14 (No 141380), and NSP15 (No 141381) [28]. Additional plasmids include: pLVX-EGFP-2xStrep (Addgene No 141395), pHAGE-N-FLAG-HA TBK1 (Addgene No 131791) IFN-Beta_pGL3 (Addgene No 102597), pNL1.1.TK [Nluc/TK] (Promega), p.GL4.45 ISRE-Luc (Promega), and p.GL4.32 NFκB-Luc (Promega). pLVX-nsp1-2xStrep containing either the K164A/H165A (NSP1^KH^) or the R125A/K126A (NSP1^RK^) mutations were generated using QuickChange site directed mutagenesis (Agilent) with the primers 5’-CTCGCGAGTAACGCCACTGCTAGCTGCCGTATTCCAGTTTTCCTGGAAA-3’ and 5’-TTTCCAGGAAAACTGGAATACGGCAGCTAGCAGTGGCGTTACTCGCGAG-3’ (KH) and 5’-CCTGCGCCCTTGTTCCCATTTGCTGCCAACAGCACCTTCCGATATGC-3’ and 5’-GCATATCGGAAGGTGCTGTTGGCAGCAAATGGGAACAAGGGCGCAGG-3’ (RK).

The sequences of all plasmids were verified by DNA sequence (Genewiz) and are available upon request. Transfections were performed using Polyjet transfection reagent (SignaGen Laboratories) according to manufacturer’s instructions.

### Immunofluorescence and quantification

HeLa cells were seeded at 1x10^5^ cells/mL into 6-well plates and transfected with 1 μg SARS CoV-2 NSP1 WT, SARS-CoV-2 NSP13, SARS-CoV-2 NSP9 or GFP the following day. Twenty-four hours post transfection, cells were infected with SeV by addition of 100 HA units/mL into medium. Six hours post-infection, cells were fixed in 4% paraformaldehyde, permeabilized, and stained. Images were acquired using a Nikon Ti2E scope. Image processing was conducted using Fiji software. Image brightness and contrast were adjusted and cells were counted using the Cell Counter plugin. Strep-tagged SARS-CoV-2 proteins were visualized and counted in the FITC (green) channel, and NF-κB protein was visualized in the TRITC (red) channel. Strep-expressing cells were selected as type 1 and cells with NF-κB-DAPI colocalization were selected as type 2. Counts were then used to calculate the frequency of type 1 to type 2.

### Antibodies

Antibodies used for immunoblot and immunofluorescence analysis include: rabbit anti-Flag (1:1000, Sigma), mouse anti-Flag M2 (1:1000, Sigma), rabbit anti-IRF3 (1:100 or 1:500, Cell Signaling Technology), mouse anti-IRF3 (1:1000, Cell Signaling Technology) rabbit anti-TBK1 (1:1000, Cell Signaling Technology), rabbit anti-phospho-TBK1 (1:1000, Cell Signaling Technology), rabbit anti-phospho-IRF3 (1:1000, Cell Signaling Technology), rabbit anti-IFIT1 (1:1000, Cell Signaling Technology), human anti-SARS-CoV-2 Spike (CR3022, 1:5000), rabbit anti-SARS-CoV-2 nucleocapsid (Genetex, 1:1000), rabbit anti-NF-κB (1:500, Cell Signaling Technology), rabbit anti-phospho-NF-κB (1:1000, Cell Signaling Technology), mouse anti-Strep (1:1000, Biolegend; 1:1000, Genscript), rabbit anti-Actin (1:1000, Cell Signaling Technology), Streptavidin-HRP (1:1000, Jackson ImmunoResearch), species-specific HRP-Conjugated antibodies (1:10,000, Jackson ImmunoResearch). Alexa Fluor conjugated secondary antibodies (1:500, Life Technologies), and nuclear counterstain 4’,6’-Diamidino-2-phenylindole dihydrochloride- (DAPI; 1:1000, ACROS Organics).

### Immunoblotting

Cells were lysed in radioimmunoprecipitation assay (RIPA) buffer (50mM Tris 10 mM 150 mM NaCl, 0.5% sodium deoxycholate, 0.1% SDS, 1% NP-40, 0.02% sodium azide) or passive lysis buffer (Promega) supplemented with cOmplete protease inhibitor cocktail (Sigma), phenylmethylsulfonyl fluoride (PMSF, Millipore), and phosphatase inhibitor (Sigma), with post-nuclear supernatants harvested by centrifugation. Protein concentration was determined by Bradford assay, and 20–30 μg quantified protein was resolved by SDS-PAGE, transferred to Polyvinylidene fluoride (PVDF) membranes using the Trans-Blot Turbo System (Bio-Rad) and blocked with either 3% bovine serum albumin (Sigma) in TBS with 0.1% Tween (TBS-T) or 5% milk in TBS-T. Membranes were probed overnight at 4°C with specific antibodies against proteins of interest, washed three times with TBS-T, incubated with species-specific HRP-conjugated antibodies (Jackson ImmunoResearch, 1:10,000), washed three times with TBS-T, and treated with Amersham ECL western blotting detection reagent (GE) or SuperSignal West Femto Substrate (Thermo Fisher). Membranes were then imaged using the Amersham Imager 680 (GE).

### Immunoprecipitation

293T cells were seeded in 10cm plates and transfected with either vector, Flag-TBK1, or co-transfected with Flag-TBK1 and SARS-CoV-2 NSP13 plasmids. Twenty-four hours post transfection, cells were harvested and lysed in IP lysis buffer (150mM NaCl, 50mM Tris pH7.4, 1mM Ethylenediamine tetraacetic acid (EDTA), 0.5% NP-40). Protein lysate was incubated with StrepTactin resin beads (IBA) and rotated with head-to-tail rotation overnight at 4°C. The next day, protein-bound beads were washed three times with IP lysis buffer and eluted from the StrepTactin resin with Buffer E (IBA). Eluates were analyzed by immunoblotting as described above.

### Promoter luciferase assays

HeLa cells seeded at 1x10^5^ cells/mL in 12-well or 24-well plates were co-transfected with pNL1.1.TK[Nluc/TK] driving NanoLuc activity, an ISRE-promoter, an IFN-β promoter, or NF-κB promoter driving firefly luciferase activity, SARS-CoV-2 viral proteins (NSP13, NSP1, etc.) or GFP, or Flag-TBK1. Transfections were completed with PolyJet (SignaGen) per manufacturer’s instructions. For assays using SeV as the immune stimulus, 100 HA units/mL were added into media for 16 hours. Cells were harvested at 16 or 24 hours post-transfection, lysed in passive lysis buffer (Promega), and assayed using a Dual Luciferase Reporter Assay System according to manufacturer’s instructions (Promega). Firefly luciferase activity was normalized to NanoLuc activity to account for transfection efficiency. Results represent at least 3 biological replicates.

### Click-iT analysis

To measure nascent protein synthesis, HeLa cells were plated into 6-well plates and transfected with 1 μg SARS-CoV-2 NSP1 WT, SARS-CoV-2 NSP1 mutant plasmids or SARS-CoV-2 NSP9 the following day. Twenty-four hours post transfection, the existing media was depleted and replenished with methionine-free DMEM (Gibco) supplemented with L-Cysteine (Alfa Aesar) for one hour. For multiplexes with immunofluorescence, cells were incubated with 100 μM homopropargylglycine (HPG) for 2 hours. Cells were washed once with PBS and then fixed with 4% paraformaldehyde. The Click-iT reaction (Thermo Fisher Scientific) was performed according to the manufacturer’s protocol and immunofluorescence performed as described above. For blots, following methionine depletion, cells were incubated with 50 μM of L-azidohomoalanine (AHA) for one hour. Cells were washed three times with PBS, harvested by centrifugation, and lysed with AHA lysis buffer (1% SDS in 50 mM Tris-HCL, pH 8.0) supplemented with protease inhibitor and 1 U/ml Benzonase Nuclease (Sigma). Protein concentration was determined by Bradford reagent. The Click-iT reaction was performed according to the manufacturer’s protocol with 40–50 μg of total protein. Lysates were run on SDS-PAGE gel as described above.

### Statistical analysis

One way ANOVA or two way ANOVA were implemented for statistical analysis of the data using GraphPad Prism software. Graphed values are presented as mean ± SD (n = 3 or greater); *p ≤ 0.05, **p ≤ 0.01, ***p ≤ 0.001 and ****p ≤ 0.0001.

## Results

### SARS-CoV-2 infected cells display limited IRF3 and NF-κB nuclear translocation during early time points of infection

**Fig 1:**
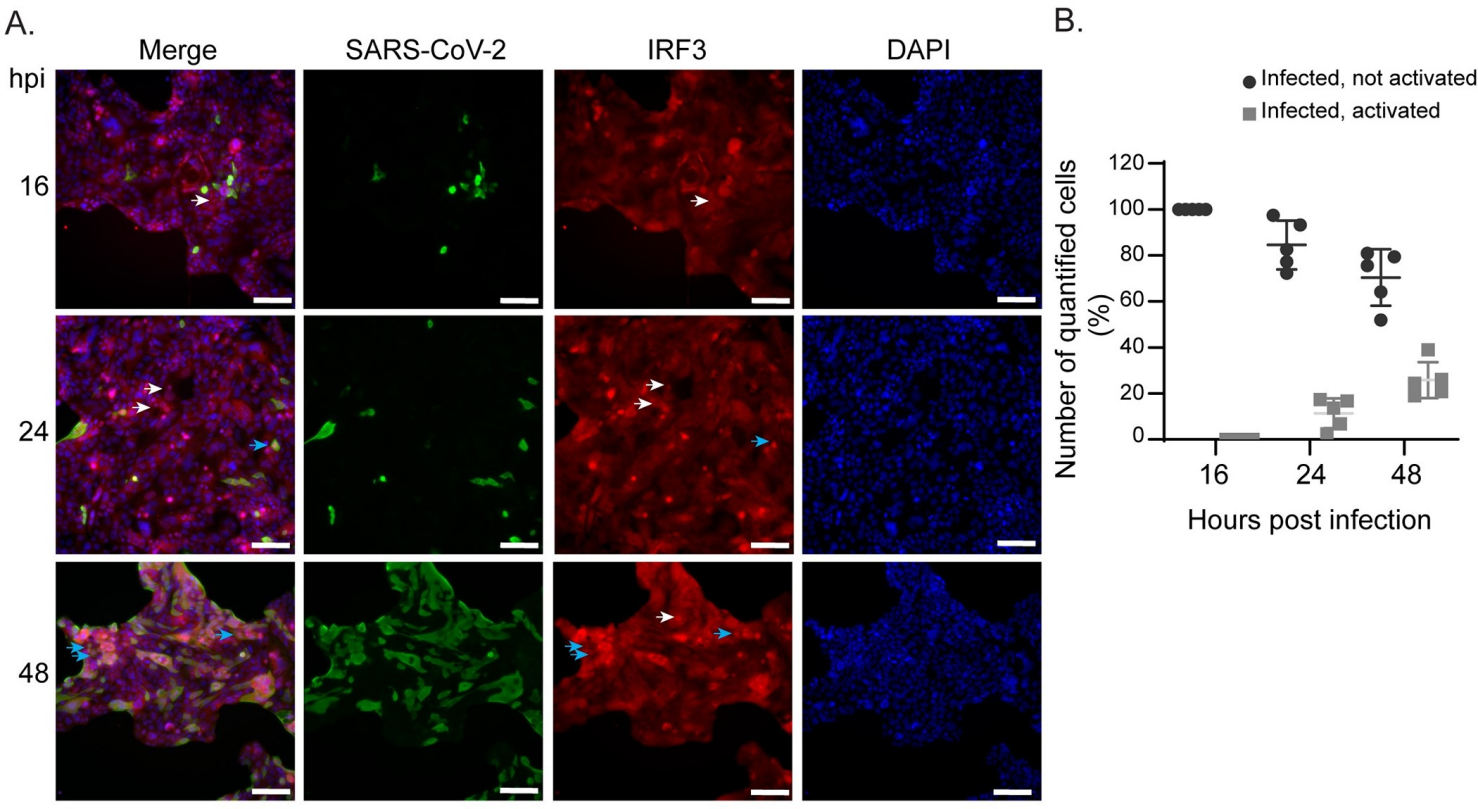
IRF3 nuclear translocation during SARS-CoV-2 infection of Calu-3 lung epithelial cells. **(A)** Representative image of Calu-3 cells at 16, 24, and 48 hours post SARS-CoV-2 infection (MOI 0.5) immunostained with anti-nucleocapsid SARS-CoV-2 (green) and anti-IRF3 (red). Nuclei are counterstained with DAPI (blue). White arrows point to neighboring cells that are activated for IRF3 transcription. Light blue arrows point to infected cells that are activated for IRF3 transcription. Scale bar: 100 μm. **(B)** Quantification of images from (A) with cells positive for SARS-CoV-2 that had nuclear translocation of IRF3. Data are displayed as mean ± SD (n = 5 images counted with at least 50 cells per image). Data were analyzed by two-way ANOVA; ****p < 0.0001, ***p < 0.001.

Previous studies have found that SARS-CoV-2 infection elicits a type I interferon response, albeit to differing amounts [22, 25, 29]. To assess the ability of SARS-CoV-2 to trigger immune activation in infected cells, we exposed Calu-3 lung epithelial cells, one of the most permissive cell lines for infection, to SARS-CoV-2 during a time course of infection. At 16, 24, and 48 hours post-infection (hpi), cultures were fixed, permeabilized, and stained for viral nucleocapsid protein to designate SARS-CoV-2 infected cells. Upon RNA virus infection, immune signaling is activated through a multi-step process where pattern recognition receptors recognize intracellular infection resulting in the nuclear translocation of the basally cytoplasmic antiviral transcription factors, IRF3 and NF-κB, an indicator of immune activation [30]. To determine if infected cells were able to block this nuclear translocation, we co-stained for IRF3 (Fig 1A). Nuclear counterstain was used to evaluate nuclear translocation of IRF3 through determining colocalization of DAPI signal with IRF3 in infected cells (Fig 1A). At 16hpi, we found that most infected cells did not cause IRF3 to translocate to the nucleus; instead, we observed this localization change at 48hpi, where approximately 25% of infected cells displayed nuclear localized IRF3 (Fig 1A; white arrows—infected cells without nuclear IRF3, blue arrows—infected cells with nuclear IRF3, quantification Fig 1B). Moreover, we also stained infected cells at 30hpi for the viral Spike protein and contrary to those cells expressing nucleocapsid protein, we observed IRF3 and also NF-κB in the nucleus in cells located adjacent to infected cells, in which limited-to-no viral antigen was detected (S1 Fig). These observations indicate productively infected SARS-CoV-2 cells have limited IRF3 and NF-κB nuclear translocation at 16hpi, but are able to activate some IRF3 at later time points, suggesting a temporal regulation of IFN activation during SARS-CoV-2 infection.

**Fig 2:**
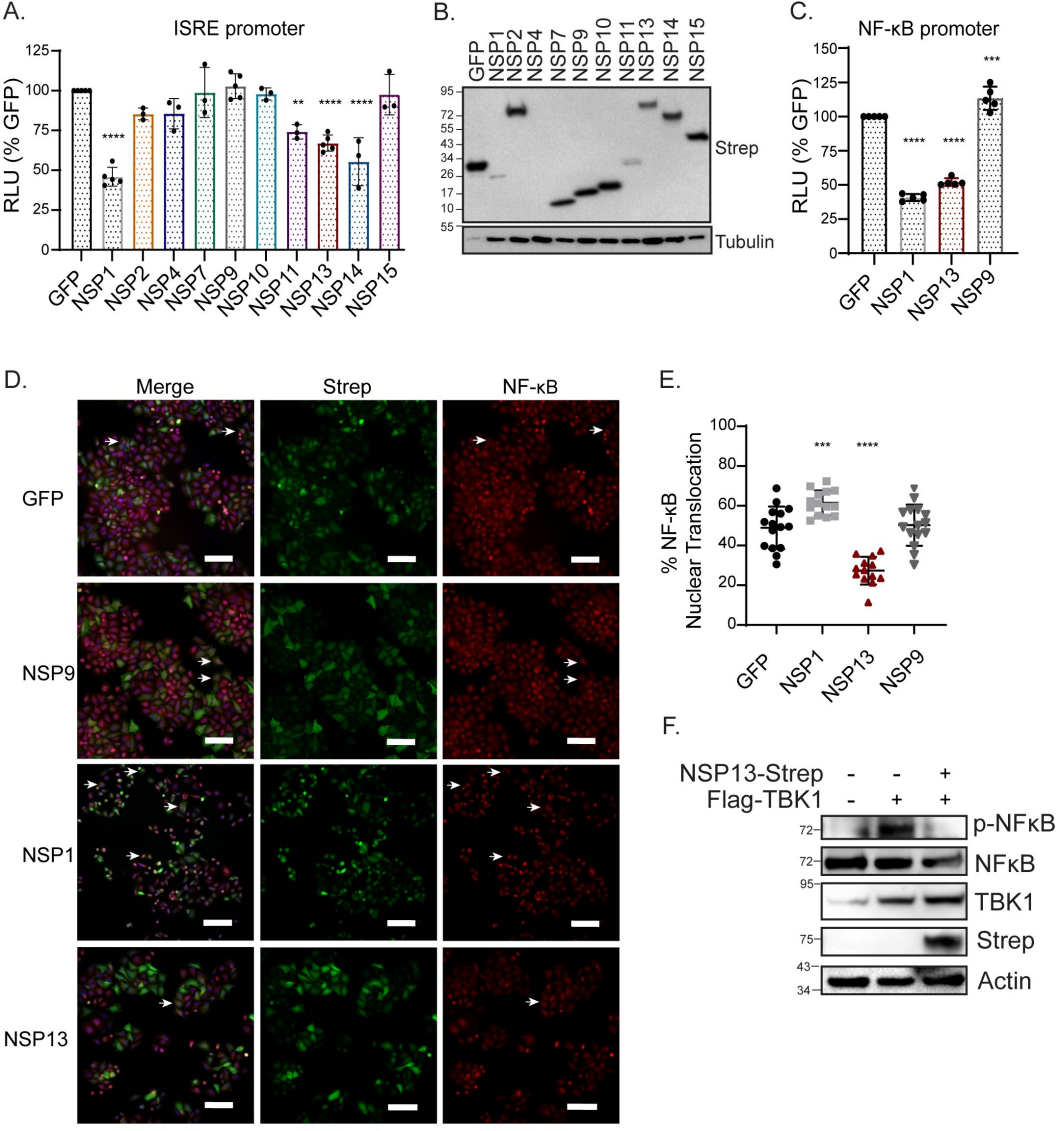
SARS-CoV-2 viral proteins NSP1 and NSP13 limit IFN response promoter and NF-κB activation. **(A)** Relative ISRE-promoter driven luciferase values of indicated viral proteins and GFP control in HeLa cells 16 hours post-SeV infection. Values depict mean ± SD (n = 3–5 biological replicates) in relative luciferase units (RLU). Data was analyzed by one-way ANOVA; **p < 0.001, ***p < 0.0001. **(B)** Immunoblot analysis of lysates harvested at 24 hours from HeLa cells transfected with the indicated Strep-tagged SARS-CoV-2 plasmids or GFP. Tubulin is shown as a loading control. Ladder is included as kDa (**C)** Relative NF-κB-promoter driven luciferase values of indicated viral proteins and GFP control in HeLa cells 16 hours post-SeV infection. Values depict mean ± SD (n = 5 biological replicates) in relative luciferase units (RLU). Data were analyzed by one-way ANOVA; **p ≤ 0.001, and ***p ≤ 0.0001. **(D)** Representative images of HeLa cells expressing the indicated viral proteins and GFP control were stimulated with SeV infection for 6 hours. Cells were immunostained for anti-NF-κB (red), anti-Strep (green) to identify cells that were expressing Strep-tagged proteins, with nuclei counterstained for DAPI (blue). White arrows point to cells where nuclear translocation of NF-κB can be observed. Scale bar: 100 μm. **(E)** Quantification of images with percent of cells positive for Strep-tag that had nuclear translocation of NF-κB. Data are displayed as mean ± SD (n = 3 biological replicates with 3–5 images per replicate counted). Data were analyzed by one-way ANOVA; ***p < 0.001, ****p < 0.0001. **(F)** Immunoblot analysis of lysates harvested at 24 hours from HeLa cells transfected with Flag-TBK1 or Flag-TBK1 and NSP13-Strep, Actin is showing as a loading control, and the ladder is included as kDa.

Our data indicate that SARS-CoV-2 can limit interferon activation. To determine the SARS-CoV-2 viral proteins that interfere with the interferon response, we screened 10 of the 16 nonstructural proteins for their ability to limit interferon stimulated response element (ISRE) activation. Sendai virus (SeV) is a known RIG-I-mediated interferon response activator [31], and thus, we stimulated HeLa cells with SeV and measured ISRE promoter activity through an ISRE-driven luciferase reporter assay. Here, we graphed the relative luciferase units (RLU) as a percentage of a GFP control plasmid and set the RLU obtained in the GFP conditions as 100%. We found that 4 out 10 viral proteins (NSP1, NSP11, NSP13, and NSP14) significantly inhibited the SeV-mediated interferon response, with a reduction of between 25–60% luciferase activity, and with the exception of NSP1 and NSP11, have similar protein expression (Fig 2A and 2B). We proceeded with NSP1 and NSP13 for further immune induction characterization and used NSP9 as a viral protein control as it did not elicit any effect on IFN response (Fig 2A). In addition to inhibiting ISRE promoter activity, NSP1 and NSP13 significantly inhibited SeV-mediated NF-κB promoter activity by approximately 2.5-fold and 2-fold, respectively (Fig 2C). To further affirm restriction of NF-κB promoter activity, we additionally used immunofluorescence and image quantification to investigate antagonism of NF-κB nuclear translocation. While NSP13 significantly inhibited both NF-κB promoter activity and nuclear translocation, rather surprisingly, we found that NSP1 instead had significantly enhanced NF-κB nuclear translocation (Fig 2D and 2E). To further define the role of NSP13 in blocking NF-κB activation, we wanted to directly activate NF-κB using the kinase TBK1. We stimulated HeLa cells with addition of exogenous TBK1 and asked whether NSP13 could block NF-κB activation, as measured by phospho-NF-κB (p-NF-κB) protein levels on an immunoblot. We found that in TBK1 only transfected cells, p-NF-κB was present, but this band was reduced when NSP13 was added, further supporting the role of NSP13 in limiting NF-κB activation (Fig 2F). Since promoter luciferase activity measures a transcription event, distinct from nuclear translocation, we deduced that inhibition must occur after nuclear translocation of transcription regulatory factors, but prior to production of the luciferase signal (i.e. at the level of transcription or translation). Additionally, while NSP13 inhibited NF-κB nuclear translocation, NSP1 did not, suggesting that NSP1 antagonizes a step in the interferon activation pathway proceeding nuclear translocation. As there is precedence for MERS and SARS-CoV NSP1 to inhibit translation [17, 19], we next asked whether SARS-CoV-2 NSP1 inhibits translation.

### SARS-CoV-2 NSP1 thwarts nascent protein synthesis

**Fig 3:**
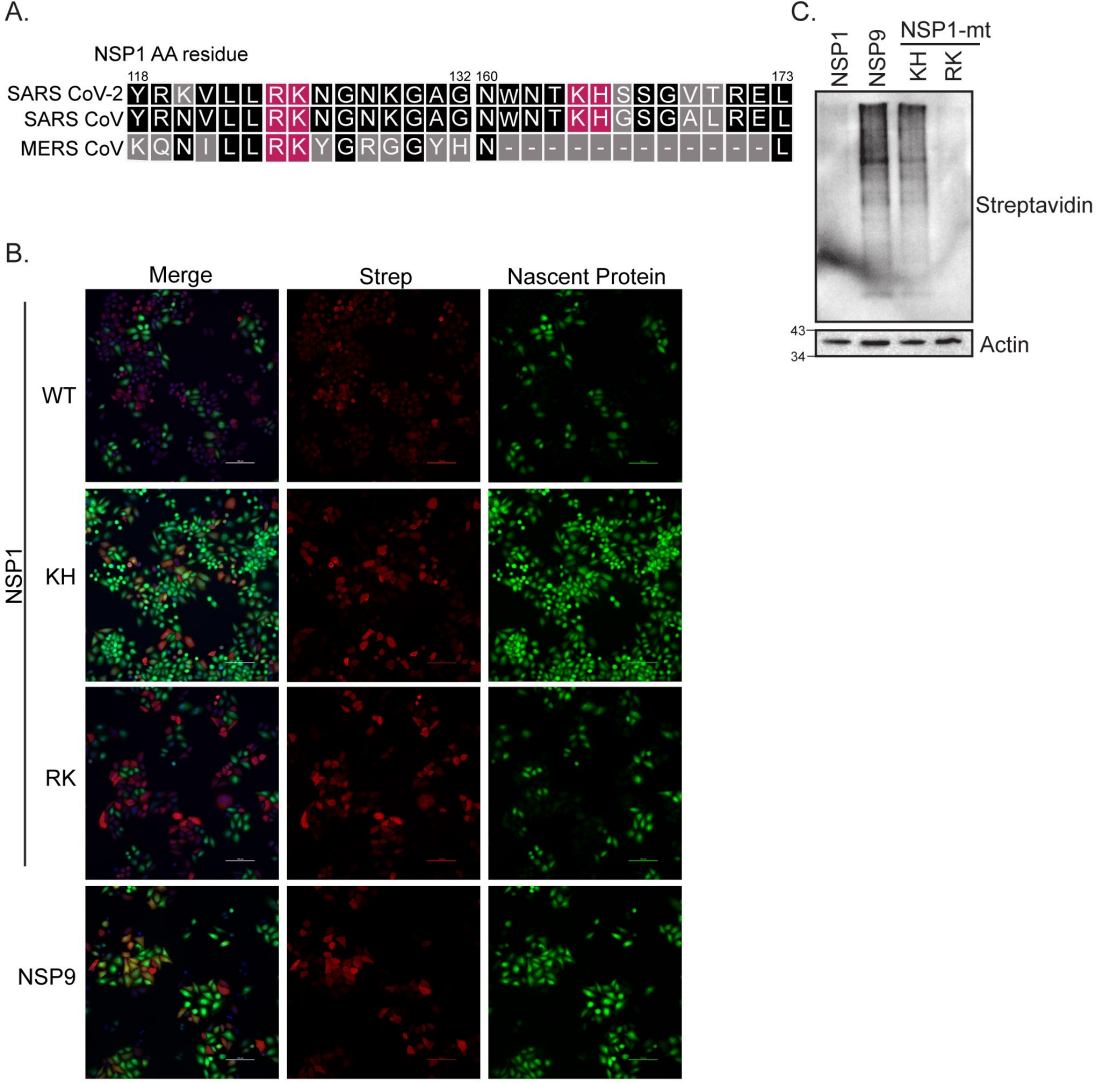
SARS-CoV-2 protein NSP1 inhibits nascent host translation. **(A)** Depiction of amino acid sequence alignment of SARS-CoV-2 NSP1, SARS-CoV NSP1, and MERS-CoV NSP1 using T-Coffee [34]. Shading depicts degree of residue conservation, with black shading indicating higher conservation. Residues mutated are indicated in pink. **(B)** Representative images of HeLa cells fixed 24 hours after metabolic-labeling with homopropargylglycine (HPG) chase. Click-chemistry was used to label incorporated Methionine-HPG in nascent proteins using Alexa-488 azide (green). Samples were then immunostained for anti-Strep (red) to identify cells specifically expressing wild-type SARS-CoV-2 NSP1, NSP9, and NSP1 mutants RK and KH. Scale bar: 100 μm. **(C)** Representative immunoblot of extracts of HeLa cells expressing Strep-tagged SARS-CoV-2 proteins NSP1 and NSP9 along with SARS-CoV-2 NSP1 mutants RK (R124/K125A) and KH (K164/H165A) 24 hours post metabolic-labeling with L-azidohomoalanine (AHA) chase, click-chemistry to incorporate HRP and ultimately visualized through anti-streptavidin-HRP blot. Actin was used as loading control, and the ladder is shown in kDa.

Both SARS-CoV and MERS-CoV are known to inhibit host gene expression through NSP1 [17, 19, 20, 32, 33]. Based on previous literature with SARS-CoV and MERS-CoV NSP1, we aligned NSP1 protein sequences of the three related coronaviruses and noted similarities between the two key sets of amino acids identified to be especially important for function (K164/H165 and R124/K125, Fig 3A and [19, 32, 34]). K164/H165 are important for ribosomal binding, while R124/K125 are necessary for RNA cleavage [34]. We mutated these two sets of residues to alanines as this residue change has been previously described to perturb the viral antagonism function in these related coronaviruses and introduced these two sets of mutants into SARS-CoV-2 NSP1 [19, 33]. We next determined the impact of wild-type (WT) and mutant NSP1 on nascent protein synthesis using metabolic labeling and click-chemistry multiplexed with immunofluorescent staining of HeLa cells transfected with GFP, NSP9, NSP1, or NSP1 mutants. We found that new protein synthesis was abolished in cells that expressed WT NSP1 (Fig 3B). This phenotype was completely reversed with the NSP1^KH^ mutant, but not with the NSP1^RK^ mutant, suggesting that the inhibition of nascent protein production by SARS-CoV-2 is independent of its RNA cleavage ability. This finding was further substantiated with the more traditional format, using metabolic-labeling, click chemistry and western blot in HeLa cells (Fig 3C).

### SARS-CoV-2 NSP13 reduces TBK1 and IRF3 phosphorylation

**Fig 4:**
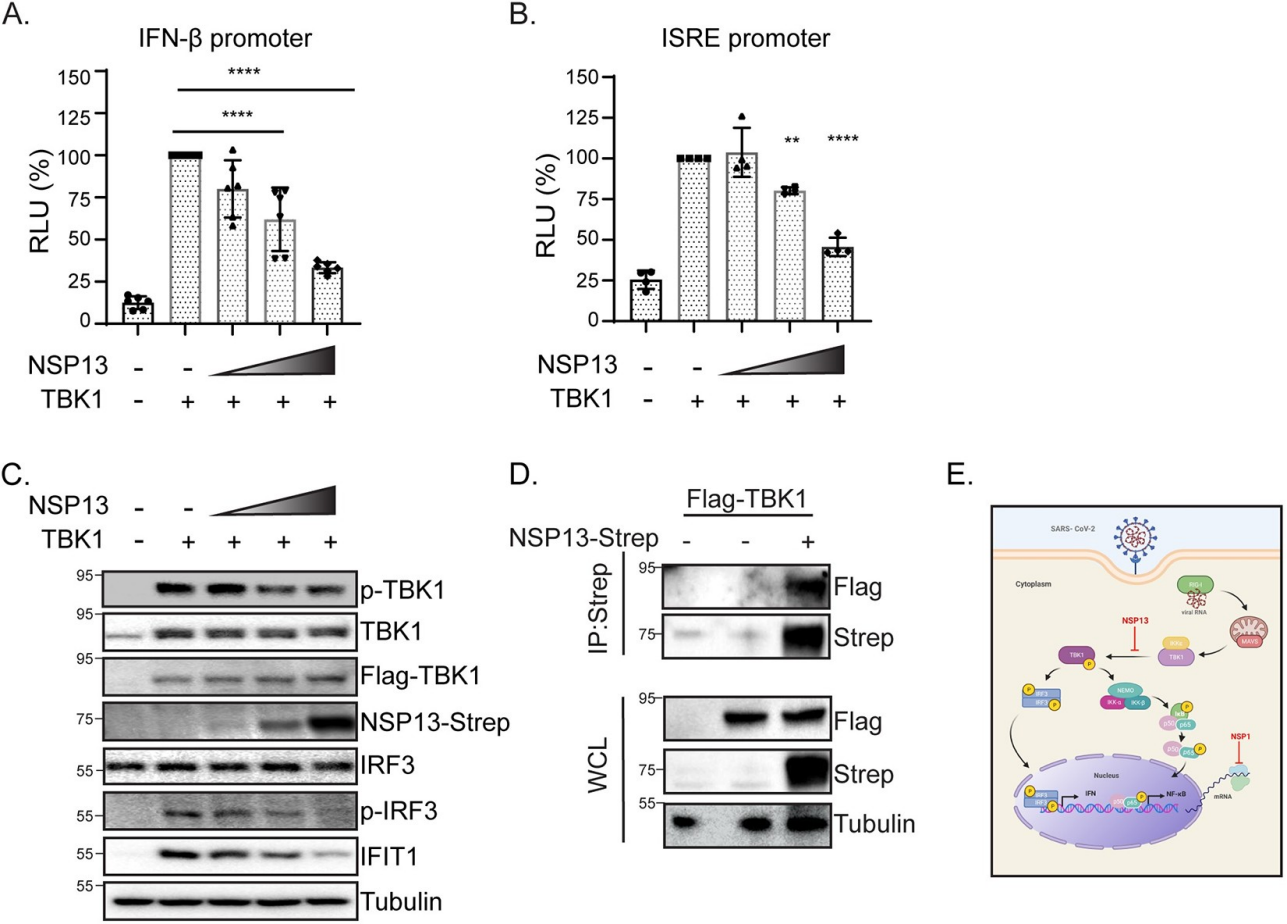
SARS-CoV-2 protein NSP13 interacts with TBK1 and blocks TBK1-mediated IFN activation. **(A)** Immunoblot analysis of anti-Strep immunoprecipitated lysates or whole cell lysate (WCL) of 293T cells transfected with Flag-TBK1 or co-transfected with Flag-TBK1 and NSP13-Strep. Actin was used as a loading control and ladder is displayed in kDa. **(B).** IFN-β-promoter driven luciferase activity of HeLa cells expressing TBK1 and varying amounts of SARS-CoV-2 NSP13 protein for 24 hours. Values depict mean ± SD (n = 6 biological replicates) in relative luciferase units (RLU). Data were analyzed by one-way ANOVA; ***p < 0.001, ****p < 0.0001 (**C**) ISRE-promoter driven luciferase activity of HeLa cells expressing TBK1 and varying amounts of SARS-CoV-2 NSP13 protein for 24 hours. Values depict the mean ± SD (n = 4 biological replicates) in relative luciferase units (RLU). Data were analyzed by one-way ANOVA; ***p < 0.001, ****p < 0.0001. **(D)** Immunoblot analysis of lysates harvested at 24 hours from HeLa cells transfected with TBK1 and varying amounts of SARS-CoV-2 NSP13. Tubulin is shown as a loading control. Ladder is included as kDa. **(E)** Schematic depicting the TBK1 pathway and effect of SARS-CoV-2 NSP1 and NSP13 on NF-κB activation and IFN-β activation. During RNA virus infection, viral RNA is recognized and bound by cytosolic recognition receptors, such as RIG-I. This binding initiates a downstream signaling cascade through the protein adaptor MAVS, which interacts with the kinases IKKε and TBK1. TBK1 phosphorylation initiates two branches of signaling: (1) dimerization and phosphorylation of the transcription factor IRF3 and (2) recruitment to a complex with the signaling molecules NEMO, IKK-α and IKK-β, subsequent phosphorylation and degradation of the inhibitor of κB (IκB), and phosphorylation of the p65 subunit of the transcription factor NF-κB. Phosphorylation of these two transcription factors results in their nuclear translocation, where they can bind to the promoters of type I IFNs and pro-inflammatory cytokines. NSP13 is shown with an inhibitory arrow at the level of TBK1 activation. NSP1 is shown with an inhibitory arrow at the level of host mRNA translation. This schematic was created using BioRender.com.

Our results suggest that NSP13 targets both IRF3 and NF-κB activation. We next sought to determine the mechanism of this targeting. TBK1 is a regulatory kinase that functions in multiple innate immune pathways and can signal through both IRF3 and NF-κB. NSP13 has been reported to bind to TBK1 [24, 28]. Given that TBK1 is involved in several signaling pathways, including IFN induction and subsequent IFN signaling, we hypothesized that NSP13 may inactivate TBK1 function, possibly by binding to TBK1, as TBK1 was predicted to be an NSP13-interacting protein in a recent protein interaction study]. To test whether NSP13 interacts with TBK1, we performed an immunoprecipitation with overexpressed NSP13 and TBK1 and found that NSP13 interacts with TBK1 (Fig 4A). We then asked whether the interaction of TBK1 with NSP13 abrogated TBK1 function. To test if NSP13 inhibited IFN activation and the downstream IFN response following TBK1 stimulus, we assessed IFN-β and ISRE-promoter activity in an NSP13-dose responsive manner. Indeed, we found that NSP13 was able to antagonize both the TBK1-induced IFN-β and ISRE promoter activity in a dose-dependent fashion (Fig 4B and 4C). To validate this observation, cell lysates from the same samples in Fig 4A were immunoblotted and probed for protein expression of the ISG, *IFIT1*, one of several TBK1-target genes [35]. We found a dose-dependent reduction in IFIT1 protein expression with increasing amounts of NSP13, phenocopying our ISRE promoter luciferase assay (Fig 4D). This suggests that NSP13 could restrict the downstream IFN response by inhibiting TBK1 phosphorylation. We observed that phospho-TBK1 (p-TBK1) and phospho-IRF3 (p-IRF3) protein levels were reduced in an NSP13-dose dependent manner, suggesting that NSP13 can limit TBK1 and IRF3 activation (Fig 4D). Taken together, these data suggest that NSP13 may limit TBK1 activation by binding to it and possibly interfering with TBK1 downstream function.

## Discussion

Our results highlight that SARS-CoV-2 employs distinct mechanisms to evade immune activation. Here, we show (1) during SARS-CoV-2 infection, IRF3 and NF-κB nuclear translocation is blocked in infected cells at early time points of infection (Fig 1 and S1 Fig); (2) the host shutoff factor, nonstructural protein 1 (NSP1) blocks IFN and NF-κB promoter activation but is unable to block SeV-mediated NF-κB nuclear translocation (Fig 2); (3) this apparent discrepancy is likely due to the ability of NSP1 to shutdown mRNA translation, which may allow for NF-κB nuclear translocation while limiting NF-κB promoter activation (Fig 3); (4) the viral helicase NSP13 evades IFN and NF-κB responses at the level of TBK1 activation (Fig 4).

Several groups have recently noted that the NSP1 residues K164 and H165 are important for protein translation inhibition for both SARS-CoV-2 and SARS-CoV [20, 24]. Additionally, Xia and colleagues very recently found that NSP13 inhibits TBK1 phosphorylation and subsequent IFN-induction and signaling [24]. Our study adds to this growing literature of SARS-CoV-2 immune evasion mechanisms by identifying that in addition to blocking IRF3-mediated IFN activation, NSP13 is also capable of obstructing NF-κB activation, suggesting that NSP13 may play a role in regulating multiple innate immune pathways during SARS-CoV-2 infection.

Our study explores immune activation, using IRF3 and NF-κB as a read-out, in the context of full virus infection. SARS-CoV-2 has several proteins that have been shown to block IFN induction [21, 24, 25]. It remains to be seen how these proteins coordinate inhibition of immune activation and which features of each viral protein guide these immune functions. NSP13 may have a role in blocking NF-κB responses that is independent of its interaction with TBK1. Indeed, a previous study identified that NSP13 may interact with TLE1 [28], a known NF-κB signaling interactor [36]. Additionally, our study did not examine downstream NF-κB-dependent gene expression in the context of NSP1 overexpression. It is possible that the reduction in downstream NF-κB promoter activity yet increased nuclear translocation during NSP1 overexpression may be due to blocking induction of these NF-κB-dependent genes.

Our study examined NSP13 function during overexpression. NSP13 is a member of the viral replication complex for SARS-CoV [37] and SARS-CoV-2 [38, 39]. We did not examine whether NSP13 localization extends beyond that of the replication complex in the context of viral infection. Thus, single protein overexpression may not fully recapitulate what is observed during full-length virus infection. Examination of the mechanisms utilized by SARS-CoV NSP13 may inform studies on SARS-CoV-2 NSP13 research as the amino acid sequences of both viral NSP13 proteins are nearly identical and diverge at only one amino acid residue [40]. Future research should be aimed at exploring other NSP13-protein interactions that may dictate immune responses and potential disease outcomes during SARS-CoV-2 infection. We have shown a role of NSP13 in blocking TBK1 activation, downstream IRF3 activation, and IFIT1 protein expression (Fig 4). Interestingly, while the protein expression levels of both phospho-IRF3 and IFIT1 are drastically reduced, the difference in protein expression levels of phospho-TBK1 is not as stark. Last, our study did not examine the role of NSP13 in limiting interferon signaling through the JAK/STAT pathway, and thus we cannot eliminate the possibility that NSP13 may be limiting both IFN-β induction and signaling. To this point, a recent study by Lei and colleagues has found that indeed NSP13 plays a role in both branches of the RIG-I-mediated innate immune pathway [25].

Recently published papers have provided structural and mechanistic insight into NSP1 translation inhibition by defining the NSP1 residues that govern this translation inhibition, that NSP1 binds to the 40S ribosomal subunit, and these events lead to subsequent immune evasion by reducing interferon mRNA expression [21, 26]. Our work supports the role of NSP1 in inhibiting translation using Click-iT chemistry [41], an assay that further validates and complements those used in these studies. It is worth noting that while our study suggests that the mechanism of translation inhibition is likely through ribosomal binding, our data do not address the exact role of the enzymatic activity of NSP1 in reducing nascent protein synthesis. Instead, however, work done by several other groups, has shown that NSP1 acts by ribosomal binding [21, 23, 26]. Future studies should examine the RNA cleavage capacity of NSP1 and whether that affects subsequent protein translation.

We did not explore the role of all SARS-CoV-2 proteins in immune signaling evasion. It is possible other SARS-CoV-2 proteins can redundantly bypass IRF3 and NF-κB responses. Indeed, Xia and colleagues describe that several viral proteins, including the M protein, ORF6, and ORF7a inhibit type I IFN signaling [24]. It is known that the SARS-CoV M protein suppresses the antiviral response, and thus SARS-CoV-2 may likely antagonize antiviral signaling through similar mechanisms [42]. Taken together, our research defines a role for the SARS-CoV-2 NSP1 and NSP13 viral proteins in evading host interferon activation through distinct mechanisms.

## Supporting information

S1 Fig IRF3 and NF-κB nuclear translocation during SARS-CoV-2 infection of Calu-3 lung epithelial cells. **(A, C)** Representative image of Calu-3 cells 30 hours post SARS-CoV-2 infection that were immunostained with anti-Spike SARS-CoV-2 (green), anti-IRF3 (A, red), or anti-NF-κB (C, red). Nuclei are counterstained with DAPI (blue) with nuclear translocation of IRF3 (A) or NF-κB (C) visualized through co-localization of DAPI signal with IRF3 (A) or NF-κB (C). White arrows point to neighboring cells that have nuclear translocation of IRF3 (A) or NF-κB (C). Light blue arrows point to infected cells that are activated for IRF3 transcription (A) or NF-κB (C). Scale bar: 50 μm. **(B)** Quantification of images from (A) with cells positive for SARS-CoV-2 that had nuclear translocation of IRF3. Data are displayed as mean ± SD (n = 5 images counted with at least 50 cells per image). Data were analyzed by one-way ANOVA; ****p < 0.0001 **(D)**. Quantification of images from (C) with cells positive for SARS-CoV-2 that had nuclear translocation of NF-κB. Data are displayed as mean ± SD (n = 5 images counted with at least 50 cells per image). Data were analyzed by one-way ANOVA; ****p < 0.0001.
(TIF)

S1 File (PPTX)
