# Supplementary figures and images for: SARS-CoV-2 viral proteins NSP1 and NSP13 inhibit interferon activation through distinct mechanisms
Source: PLoS One. 2021 Jun 24;16(6):e0253089. doi: 10.1371/journal.pone.0253089 (PMC8224853; doi:10.1371/journal.pone.0253089)

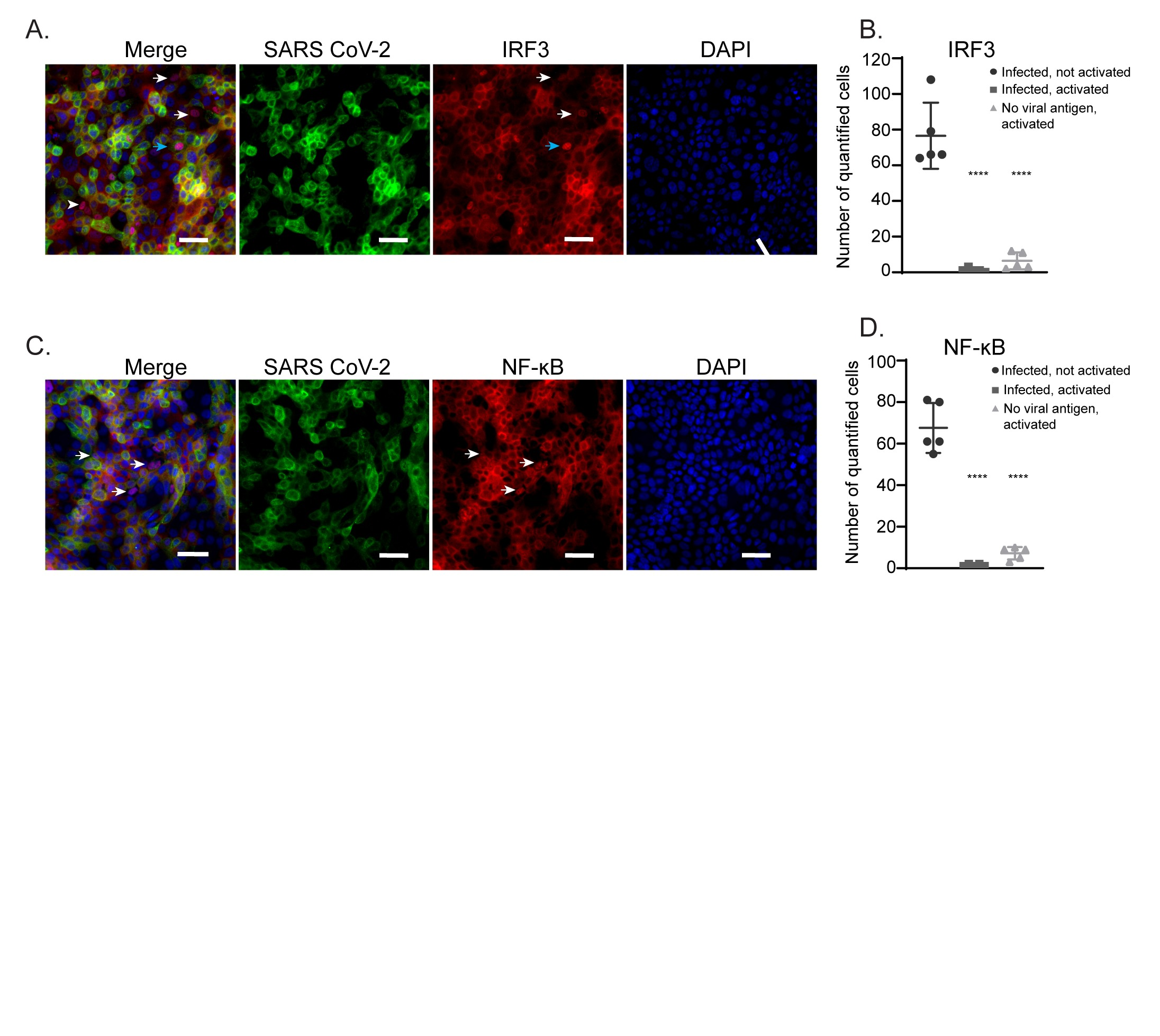

Supplement: S1 Fig — (A, C) Representative image of Calu-3 cells 30 hours post SARS-CoV-2 infection that were immunostained with anti-Spike SARS-CoV-2 (green), anti-IRF3 (A, red), or anti-NF-κB (C, red). Nuclei are counterstained with DAPI (blue) with nuclear translocation of IRF3 (A) or NF-κB (C) visualized through co-localization of DAPI signal with IRF3 (A) or NF-κB (C). White arrows point to neighboring cells that have nuclear translocation of IRF3 (A) or NF-κB (C). Light blue arrows point to infected cells that are activated for IRF3 transcription (A) or NF-κB (C). Scale bar: 50 μm. (B) Quantification of images from (A) with cells positive for SARS-CoV-2 that had nuclear translocation of IRF3. Data are displayed as mean ± SD (n = 5 images counted with at least 50 cells per image). Data were analyzed by one-way ANOVA; ****p < 0.0001 (D). Quantification of images from (C) with cells positive for SARS-CoV-2 that had nuclear translocation of NF-κB. Data are displayed as mean ± SD (n = 5 images counted with at least 50 cells per image). Data were analyzed by one-way ANOVA; ****p < 0.0001. (TIF) [file pone.0253089.s001.tif]
