# Supplementary material for: SARS-CoV-2 viral proteins NSP1 and NSP13 inhibit interferon activation through distinct mechanisms
Source: PLoS One. 2021 Jun 24;16(6):e0253089. doi: 10.1371/journal.pone.0253089 (PMC8224853; doi:10.1371/journal.pone.0253089)
Supplement: S1 File — (PPTX) [file pone.0253089.s002.pptx]

## Slide 1
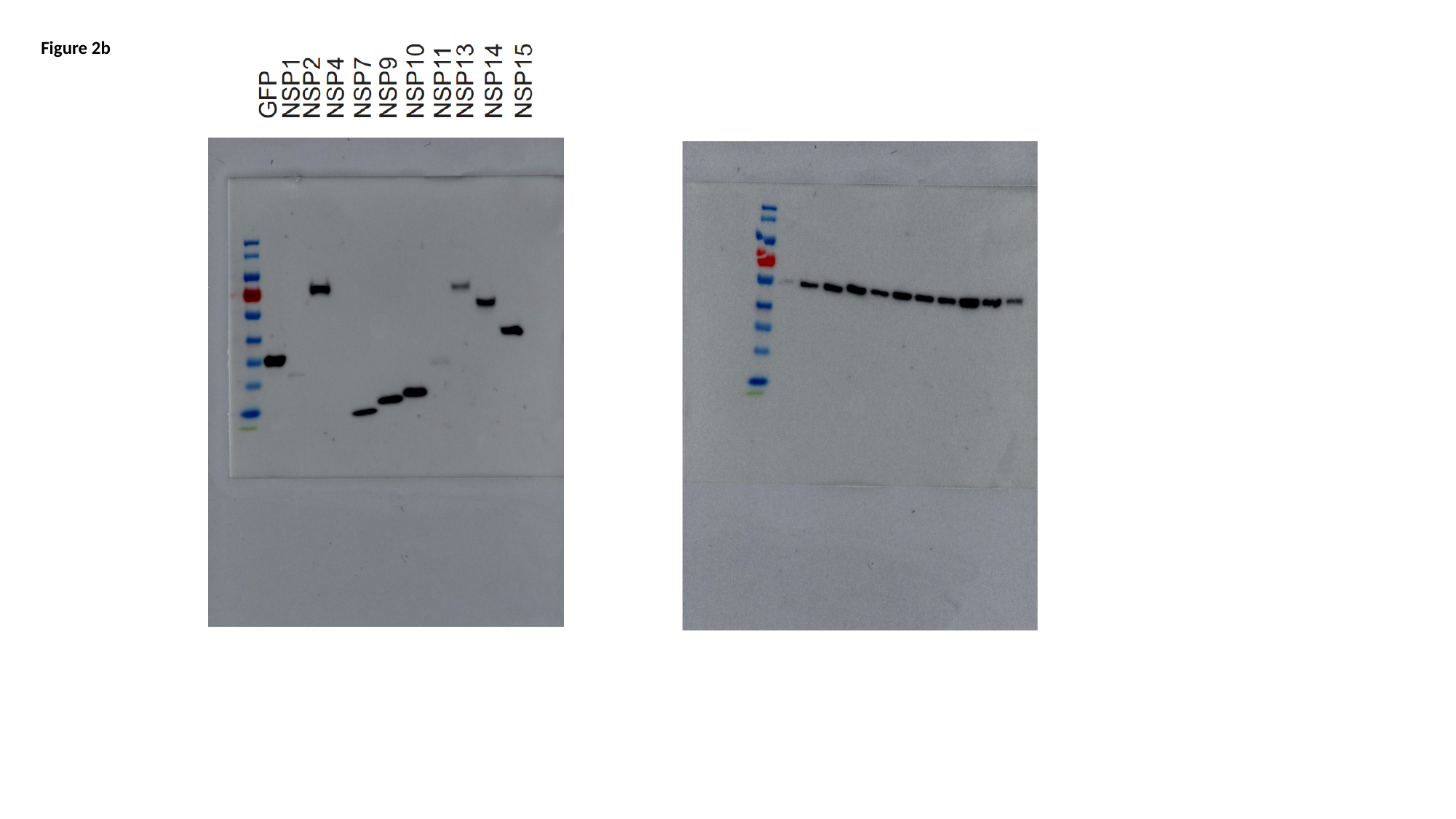

Figure 2b

## Slide 2
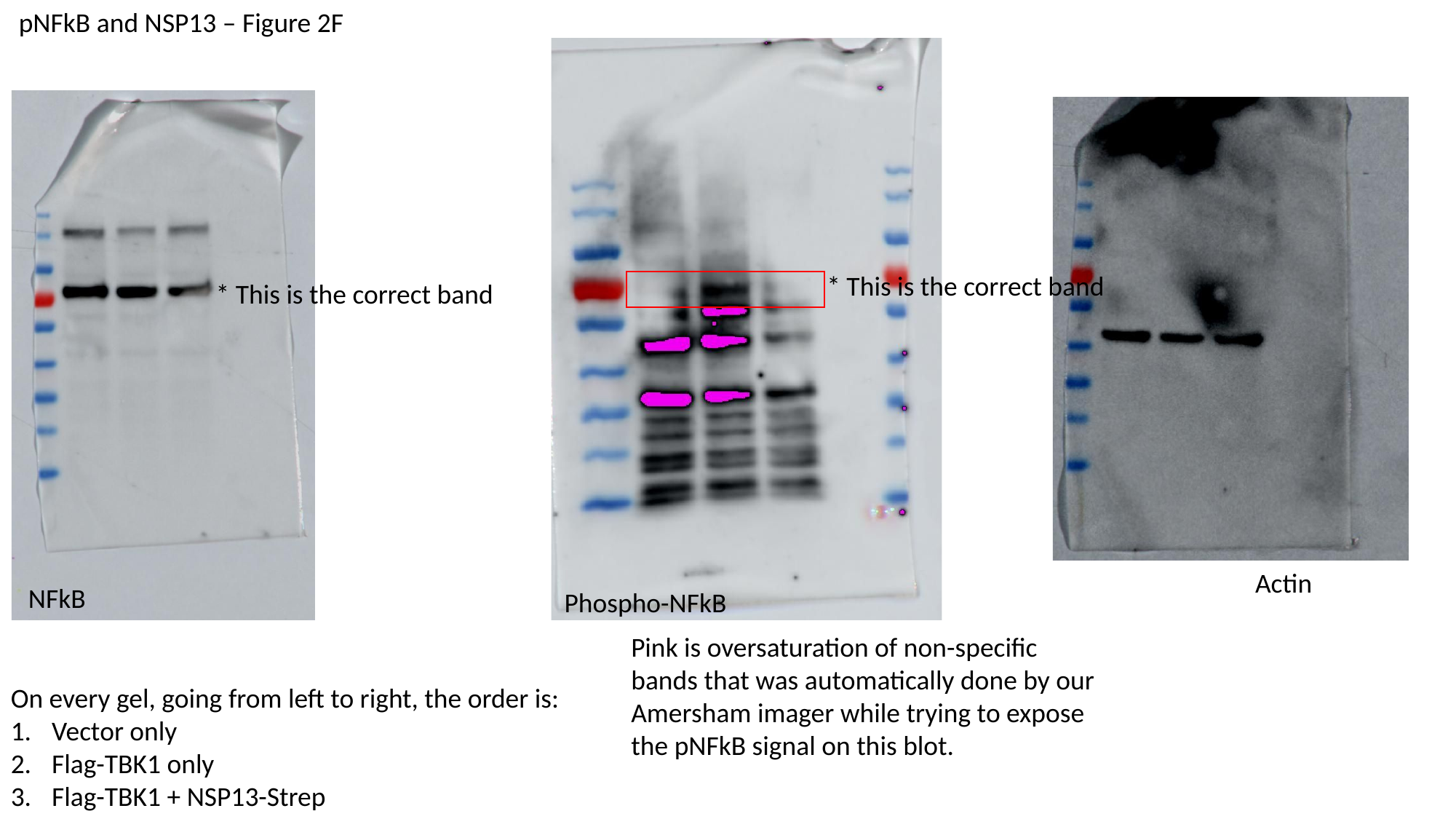

pNFkB and NSP13 – Figure 2F
*
* This is the correct band
* This is the correct band
*This is residual Strep protein
Actin
NFkB
Phospho-NFkB
Pink is oversaturation of non-specific bands that was automatically done by our Amersham imager while trying to expose the pNFkB signal on this blot.
On every gel, going from left to right, the order is:
Vector only
Flag-TBK1 only
Flag-TBK1 + NSP13-Strep

## Slide 3
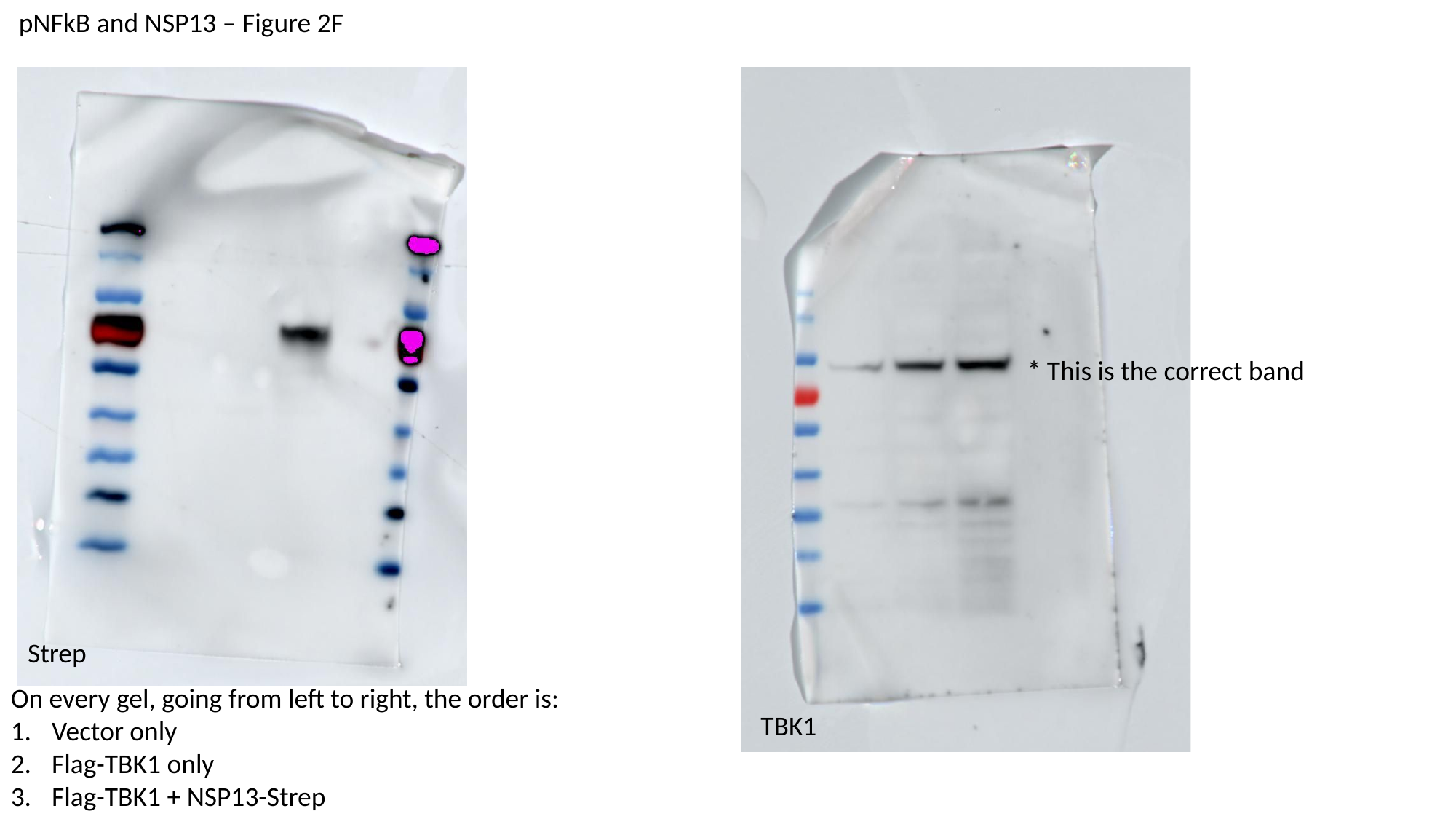

pNFkB and NSP13 – Figure 2F
* This is the correct band
Strep
On every gel, going from left to right, the order is:
Vector only
Flag-TBK1 only
Flag-TBK1 + NSP13-Strep
TBK1

## Slide 4
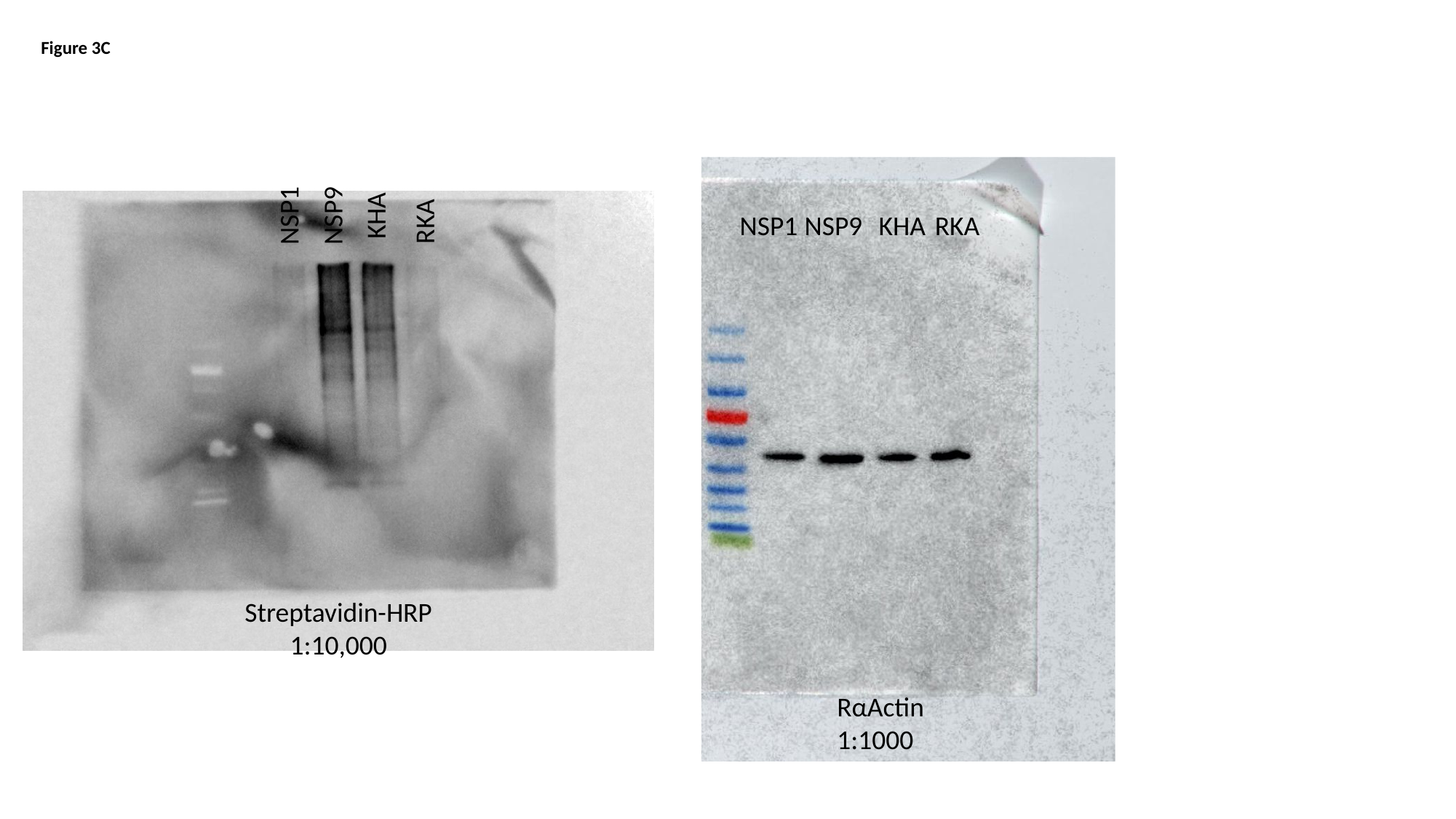

Figure 3C
NSP1
NSP9
KHA
RKA
NSP1
NSP9
KHA
RKA
Streptavidin-HRP
1:10,000
RαActin
1:1000

## Slide 5
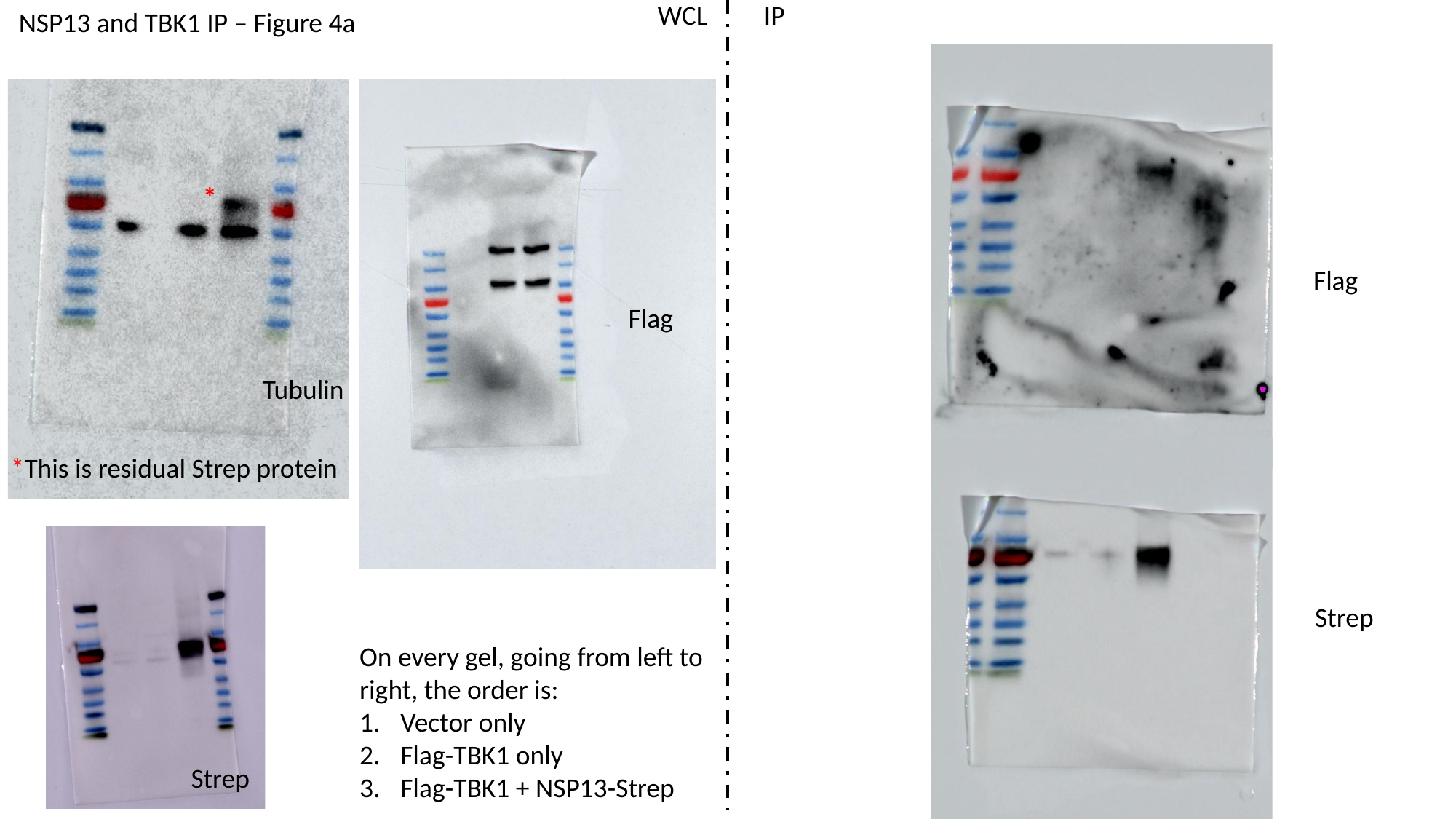

NSP13 and TBK1 IP – Figure 4a
WCL
IP
*
Flag
Flag
Tubulin
*This is residual Strep protein
Strep
On every gel, going from left to right, the order is:
Vector only
Flag-TBK1 only
Flag-TBK1 + NSP13-Strep
Strep

## Slide 6
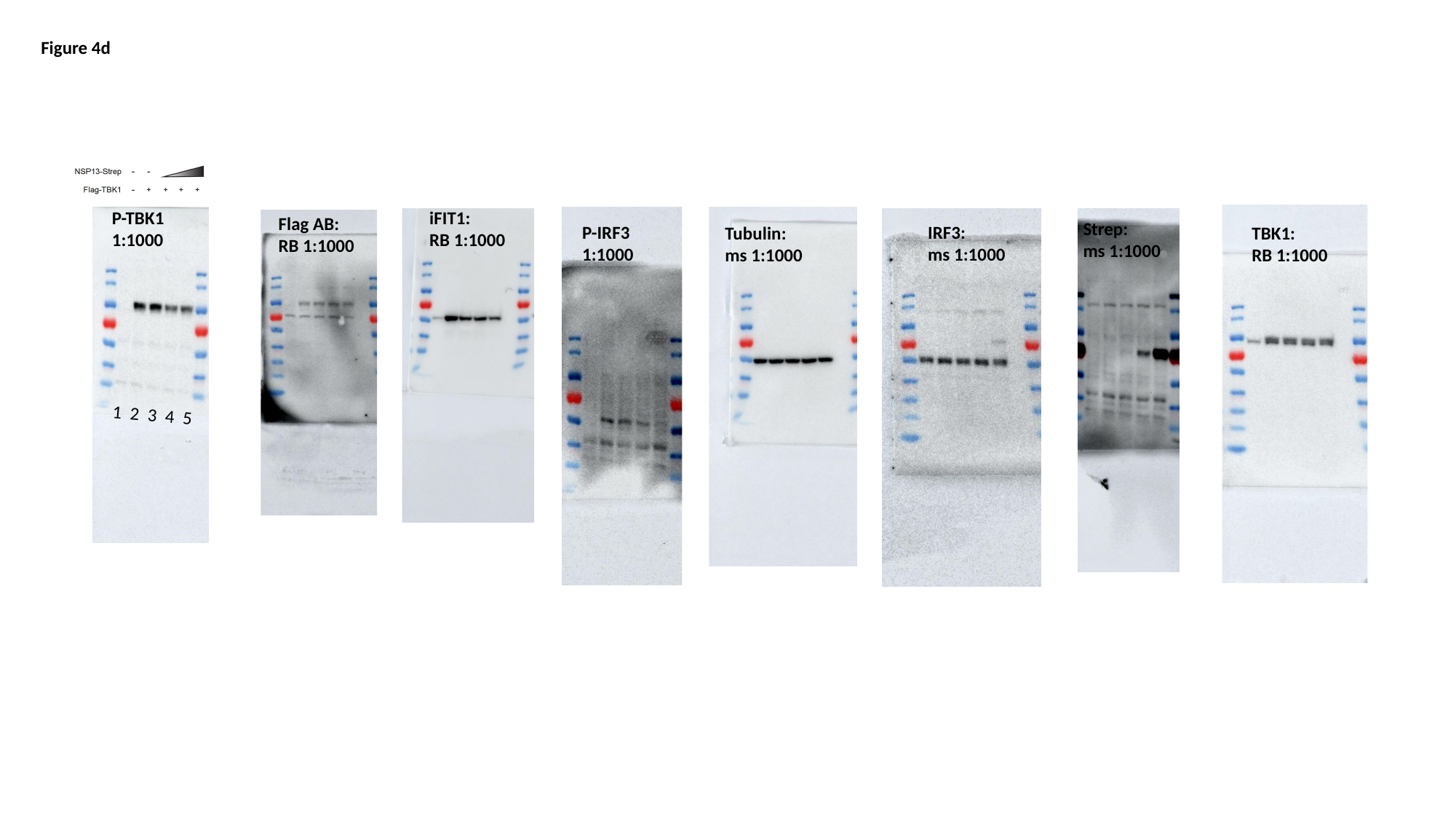

Figure 4d
P-TBK1
1:1000
iFIT1:
RB 1:1000
Flag AB:
RB 1:1000
Strep:
ms 1:1000
P-IRF3
1:1000
IRF3:
ms 1:1000
Tubulin:
ms 1:1000
TBK1:
RB 1:1000
1 2 3 4 5
